# Maternal and Neonatal Outcomes After Planned or Emergency Delivery for Placenta Accreta Spectrum: A Systematic Review and Meta-Analysis

**DOI:** 10.3389/fmed.2021.731412

**Published:** 2021-09-28

**Authors:** Wei Zhong, Fang Zhu, Shengqiong Li, Jin Chen, Fengya He, Jie Xin, Mei Yang

**Affiliations:** ^1^Department of Obstetrics, Hospital of Chengdu University of Traditional Chinese Medicine, Chengdu, China; ^2^Department of Pain, Hospital of Chengdu University of Traditional Chinese Medicine, Chengdu, China; ^3^Department of Traditional Therapy, Hospital of Chengdu University of Traditional Chinese Medicine, Chengdu, China

**Keywords:** placenta accrete, maternal outcomes, neonatal outcomes, planned delivery, emergency delivery

## Abstract

**Objective:** To compare maternal and neonatal outcomes for women with placenta accreta syndrome (PAS) delivering *via* a planned or emergent approach.

**Methods:** A systematic search for relevant studies was conducted by screening the PubMed, Scopus, Web of Science, and Google Scholar electronic databases. Included studies should have been retrospective record-based or prospective in design. They must have compared maternal and/or neonatal outcomes for PAS patients delivering *via* planned and emergency procedures. Strength of association was presented as pooled adjusted relative risk (RR) for categorical outcomes and weighted mean difference (WMD) for continuous outcomes. Statistical analysis was done using STATA version 16.0.

**Results:** Nine articles were included in the meta-analysis. PAS patients undergoing planned deliveries had increased gestational ages, required fewer units of transfused blood, experienced shorter hospital stay durations, and presented reduced risks for maternal ICU admission and severe maternal morbidity. Neonates born to mothers undergoing planned deliveries had increased birth weights and decreased NICU admission risk.

**Conclusion:** These findings indicate a planned approach for delivery is better for maternal and neonatal outcomes compared to urgent/emergency delivery for PAS patients.

## Introduction

Available evidence suggests that the incidence of placenta accreta spectrum (PAS) varies from 1 in around 250 deliveries to 1 in 500 deliveries (1, 2). Placenta accreta spectrum includes placenta accreta, placenta increta and placenta percreta, is a clinical condition wherein placenta grows too deep inside the uterus and is a potentially life-threatening condition owing to the increased likelihood of severe postpartum bleeding (3). Especially in accreta, the invasion of placental trophoblast is usually limited to myometrium; in increta, the trophoblast invades the myometrium and in percreta, invasion occurs through the myometrium to the serosa and/or adjacent pelvic organs (4, 5). In the usual course, placenta detaches from the uterus after delivery but in case of placenta accrete, a part or whole of placenta remains attached to uterus leading to massive bleeding. Ultrasonography, particularly in the second trimester, is helpful in arriving at the diagnosis of PAS (6, 7). It is very important that an early diagnosis is made and a careful planning of delivery at an advanced clinical center is done, possibly involving a multi-disciplinary team with immense expertise and experience.

A commonly adopted management strategy has been to proceed for planned delivery at 34 weeks in women with PAS with the intent to reduce the risk of bleeding (8, 9). The evidence behind this comes from a decision tree analysis published in 2010 (4, 8). However, there is a major limitation with extrapolating the findings of this decision analysis to women with PAS as the analysis was based on women with placenta previa. The International Society for PAS (IS-PAS) guidelines state that expectant management to be done until after 36 weeks of gestation in those with no prior risk factors such as preterm delivery and at around 34 weeks in those with history of previous preterm birth or recurrent vaginal bleeding or premature rupture of membranes (PROM) (10, 11). It has been really challenging to conduct deliveries in women with suspected or confirmed PAS. Studies have also shown that women with placenta accreta tend to have a higher likelihood of having delivery through cesarean section, admitted to intensive care unit and having neonate born preterm and admitted to neonatal intensive care unit (NICU) (12, 13).

There have been studies that aimed to look at and compare maternal as well as neonatal outcomes according to planned or emergent approach for management. However, there have not been any prior attempts to summarize the findings using meta-analytic methods. The current study aimed to fill this gap in evidence and compare maternal outcomes such as gestational age at delivery, estimated blood loss, number of blood units transfused, admission to intensive care unit (ICU), length of hospital stay and neonatal outcomes like birth weight, admission to neonatal intensive care unit (NICU) and APGAR score.

## Materials and Methods

### Search Strategy

This study was conducted in compliance with PRISMA (Preferred Reporting Items for Systematic Reviews and Meta-analyses) guidelines. We conducted a search of PubMed, Scopus, Web of Science and Google Scholar academic databases for relevant English language publications published prior to 10th August 2021. Search strategy used both medical subject headings (MeSH) terminology and free text words (Supplementary Table 1). This literature search aimed to identify studies comparing maternal and neonatal outcomes in PAS patients undergoing planned or emergency delivery.

### Selection Criteria and Methods

Studies of interest identified by database screening were reviewed independently by two subject experts. First, duplicates were removed. Then titles and abstracts were reviewed. Studies passing these two rounds underwent full-text review. Disputes were resolved through discussion. Studies cited by included studies were also reviewed.

#### Inclusion Criteria

To be eligible for inclusion, studies must have been retrospective record-based or prospective in design. They should have been conducted in apparently healthy mothers with no underlying major illness. Further, eligible studies should have compared and reported relevant maternal and/or neonatal outcomes for PAS patients undergoing planned and emergency deliveries.

#### Exclusion Criteria

Case reports and reviews were excluded. Studies that did not provide data on outcomes of interest or did not compare between PAS patients undergoing planned and emergency delivery were excluded. Studies focusing only on placenta previa were excluded. Further, studies that were qualitative in methodology were also excluded.

### Data Extraction and Quality Assessment

Two authors independently extracted data from included studies using a standardized guide. Extracted data included identification features (author name, year of publication), study setting, design, subject characteristics, overall sample size, and main findings. Study quality was assessed using the Newcastle-Ottawa Quality Assessment Scale (14).

### Statistical Analysis

All analyses were done using STATA version 16.0. This meta-analysis reported effect sizes as pooled relative risk values with 95% CIs (confidence intervals) for categorical outcomes and weighted mean differences (WMD) for continuous outcomes. I^2^ denoted heterogeneity. In instances where I^2^ exceeded 40%, a random effects model was used. For outcomes where the I^2^ was ≤40%, fixed effects model was used (15). *P*-values < 0.05 was considered statistically significant. Egger's test was employed to assess publication bias. We chose not to present funnel plot, as visual inspection of a funnel plot may give a misleading impression of the presence or absence of publication bias. It is preferred to use a formal statistical test for bias, such as Egger's test (16).

## Results

### Literature Screening and Selection

**Figure 1:**
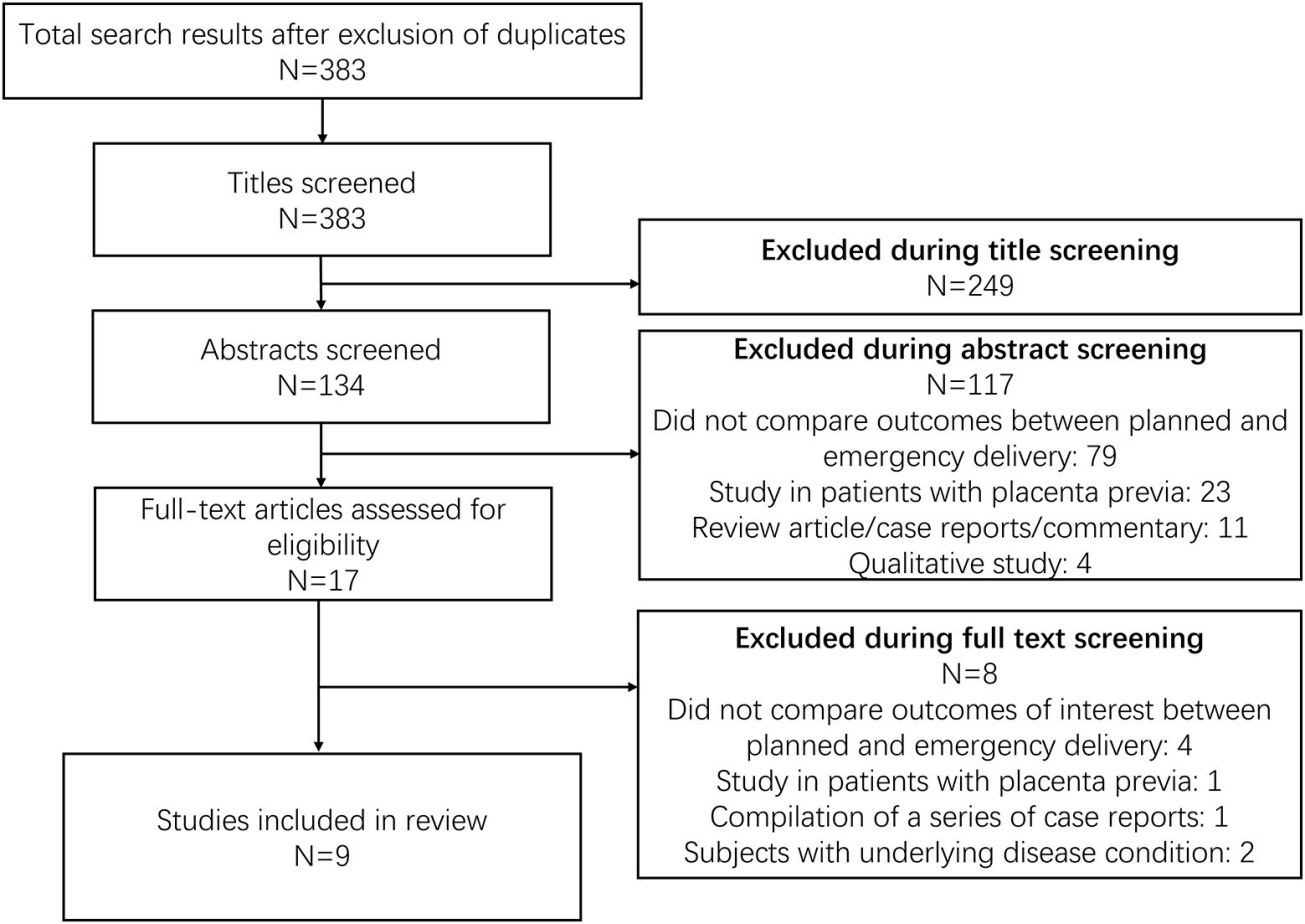
Study screening process.

Database screening returned 383 citations of interest (Figure 1). Title screening resulted in 249 removals, while abstract screening further eliminated 117 from contention. Seventeen studies were reviewed in detail, with nine meeting inclusion criteria (Table 1) (17–25). Three of these nine were conducted in the USA, two in China, one each in Lebanon, Argentina, and Israel, and one was multicentric. All studies but two were retrospective in design. Overall study quality was good (Supplementary Table 2), with most studies reporting appropriate selection of participants, controlling for baseline differences, providing reasonable explanations for follow up attrition, and giving criteria/operational definition(s) for ascertaining exposures and outcomes.

**Table 1:** Summary of included studies.

**References**	**Study design**	**Country**	**Participant characteristics**	**Sample size**	**Key outcome (Elective vs. emergent)**
Seoud et al. (17)	Prospective cohort	Lebanon	Patients with placenta accrete and mean age of 34 years and mean parity of 3.	28 (22 with elective delivery and 6 with emergent delivery)	Gestation [weeks; Mean (SD)]: 34.46 (3.15) vs. 27.38 (8.90) Total blood loss (ml) (mean, SD): 1,841 (923) vs. 3,166 (1,211) Need for transfusion: RR 0.50 (95% CI: 0.33, 0.76) Total units of blood transfused (Mean, SD): 2.18 (2.60) vs. 10.67 (6.91) Length of hospital stay (days) (mean, SD): 3.70 (0.70) vs. 5.20 (4.50) Neonatal weight in grams (Mean, SD): 2396.14 (620.40) vs. 2031.25 (396.20) Small for gestational age: RR 2.74 (95% CI: 0.17, 44.8) APGAR score: At 5 min: 9 (2) vs. 7(5) NICU admission: RR 0.41 (95% CI: 0.17, 0.99)
Fishel Bartal et al. (18)	Retrospective cohort	USA	Patients with placenta accrete and mean age of 33 years; majority had prior cesarean delivery (around 80%)	109 (68 with elective delivery and 41 with emergent delivery)	Gestation [weeks; Mean (SD)]: 33.8 (4.80) vs. 30.3 (6.2) Total blood loss (ml) (mean, SD): 2,000 (250) vs. 3,000 (566.6) Need for transfusion: RR 2.07 (95% CI: 0.98, 4.36) Total units of blood transfused (Mean, SD): 2.0 (0.67) vs. 5 (0.83) Length of hospital stay (days) (mean, SD): 4.4 (3.0) vs. 4.5 (1.6) Need for ICU admission for the mother: RR 0.45 (95% CI: 0.24, 0.86) Severe maternal morbidity: RR 0.58 (95% CI: 0.39, 0.86) Neonatal weight in grams (Mean, SD): 2446.3 (600.5) vs. 1728.8 (824) NICU admission: RR 0.87 (95% CI: 0.69, 1.09) Any neonatal complication/morbidity: RR 1.06 (95% CI: 0.58, 1.91)
Morlando et al. (19)	Combination of both retrospective data and data collected prospectively	Multi centric study (Italy, Germany, Finland, Belgium, Austria, France, Netherlands, UK)	Patients with placenta accrete and mean age of around 35 years; mean parity of 2; majority had ethnicity of European, Middle Eastern, and Latin American (around 81%); mean BMI was around 25 Kg/m^2^	356 (239 with elective delivery and 117 with emergent delivery)	Gestation [weeks; Mean (SD)]: 36 (0.33) vs. 34 (0.66) Total blood loss (ml) (mean, SD): 2,000 (466.6) vs. 1,550 (416.7) Total units of blood transfused (Mean, SD): 2.0 (1) vs. 2 (1) Need for ICU admission for the mother: RR 0.72 (95% CI: 0.55, 0.94) Severe maternal morbidity: RR 0.58 (95% CI: 0.31, 1.08) Neonatal weight in grams (Mean, SD): 3030 (105.8) vs. 2860 (58.33) APGAR score at 5 min (Mean; SD): 9 (0.33) vs. 10 (0.17) Any neonatal complication/morbidity: RR 0.84 (95% CI: 0.46, 1.53)
Kong et al. (20)	Retrospective cohort	China	Patients with placenta accrete and mean age of around 30 years; mean parity of 1; majority had associated placenta Previa (around 66%)	47 (29 with elective delivery and 18 with emergent delivery)	Gestation [weeks; Mean (SD)]: 36.1. (1.1) vs. 33.12 (0.93) Total blood loss (ml) (mean, SD): 1,500 (1183.33) vs. 2,400 (1,350) Need for ICU admission for the mother: RR 0.62 (95% CI: 0.32,1.19) Maternal length of hospital stay ≥ 7 days: RR 0.55 (95% CI 0.28,1.08) Risk of Neonatal weight < 2,000 g: RR 0.81 (95% CI:0.53,1.23) NICU admission: RR 0.66 (95% CI: 0.47, 0.93)
Pri-Paz et al. (21)	Retrospective Record based study	USA	Patients with placenta accrete (58.3%) and mean gravida of 5.6; mean parity of 2.9; majority had associated placenta previa (around 91.7%); majority were of age more than 35 years (around 55%)	48 (24 with elective delivery and 24 with emergent delivery)	Risk of GA < 37 weeks: RR 0.86 (95% CI: 0.65, 1.13) Total blood loss > 5,000 ml: RR 0.83 (95% CI: 0.45, 1.55) Risk of need of blood transfusion ≥ 5 units: RR 0.94 (95% CI:0.64,1.38) Need for ICU admission for the mother: RR 0.57 (95% CI: 0.30, 1.10) Risk of Neonatal weight less than 2.5 Kg: RR 0.23 (95% CI: 0.09, 0.57) Five minute Apgar < 7: RR 0.43 (95% CI 0.09, 2.02) NICU admission: RR 0.74 (95% CI: 0.52, 1.05)
Shamshirsaz et al. (22)	Retrospective case control study	USA	Patients with placenta accreta and mean age of around 33 years; mean parity of 1; majority had associated placenta previa (around 77%); mean gravida of 4; mean BMI of around 32 kg/m^2^	130 (70 with elective delivery and 60 with emergent delivery)	Gestation [weeks; Mean (SD)]: 34 (0.17) vs. 32 (0.5) Total blood loss (ml) (mean, SD): 1,725(300) vs. 1,600 (300) Total units of blood transfused (Mean, SD): 1.0 (0.67) vs. 3.0 (1.33) Maternal length of post-operative hospital stay (days) (mean, SD): 4.0 (0.33) vs. 5.0 (0.33) Severe maternal morbidity: RR 0.66 (95% CI:0.45,0.95) Neonatal weight in grams (Mean, SD): 2,100(400) vs. 1,400 (600) NICU admission: RR 0.84 (95% CI: 0.72, 0.98) Any neonatal complication/morbidity: RR 0.51 (95% CI: 0.37,0.71) Five minute Apgar < 7: RR 0.35 (95% CI 0.21, 0.59)
Stanleigh et al. (23)	Prospective observational study	Israel	Patients with placenta accreta and mean age of around 35 years; parity ranging from 4-6; >90% had previous cesarean delivery	72(22 with elective delivery and 50 with emergent delivery)	Gestation [weeks; Mean (SD)]: 34.1 (3.62) vs. 34.9 (2.9) Risk for Need for transfusion: RR 0.38 (95% CI: 0.12, 1.15) Total units of blood transfused (Mean, SD): 1.5 (0.67) vs. 4 (1.08) Length of hospital stay (days) (mean, SD): 10.16 (4.5) vs. 10.2 (1.6) Risk of need for hospitalization of mother > 7 days: RR 1.14 (95% CI: 0.64, 2.01) Severe maternal morbidity: RR 0.59 (95% CI: 0.30, 1.14) Neonatal weight in grams (Mean, SD): 2317 (798.9) vs. 2460 (682.9) Risk of Neonatal weight less than 2.5 Kg: RR 1.81 (95% CI:1.02,3.21) Five minute Apgar < 7: RR 1.26 (95% CI 0.47, 3.33)
Wang et al. (24)	Retrospective cohort study	China	Patients with placenta accreta and mean age of around 34 years; mean parity of one; around 45-50% had previous history of cesarean delivery	180 (126 with elective delivery and 54 with emergent delivery)	Gestation [weeks; Mean (SD)]: 36.7 (1.9) vs. 36.7(2.4) Total blood loss (ml) (mean, SD): 830 (266.6) vs. 1,000 (287.5) Need for ICU admission for the mother: RR 0.86 (95% CI: 0.48, 1.54)
Meller et al. (25)	Retrospective cohort	Argentina	Patients with placenta accrete; around 60% had previous history of cesarean delivery	81 (50 with elective delivery and 31 with emergent delivery)	Gestation [weeks; Mean (SD)]: 36.2 (1.0) vs. 30.7 (1.3)

### Maternal Outcomes

**Figure 2:**
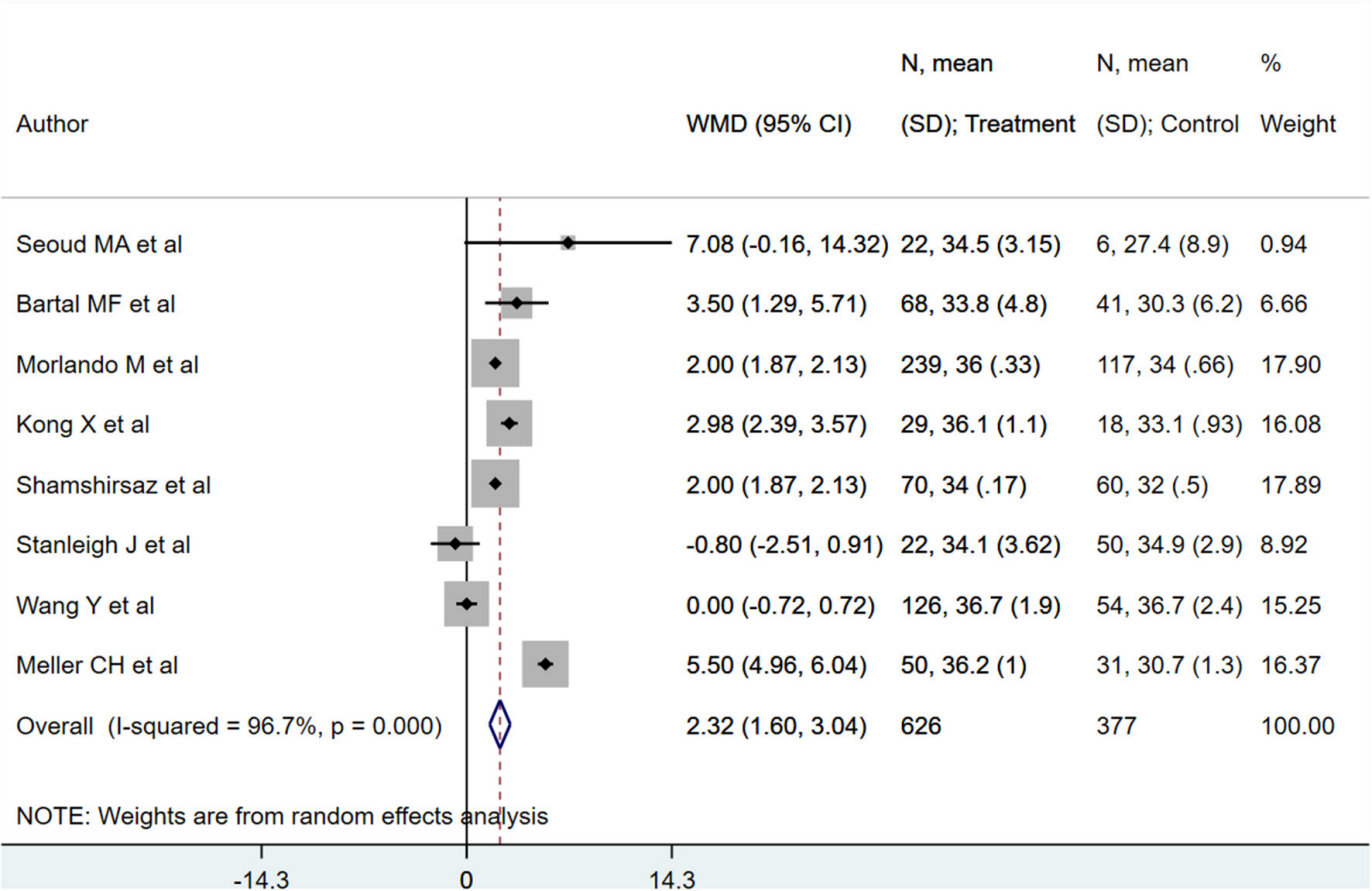
Gestational age for PAS patients undergoing planned or emergency delivery.

**Figure 3:**
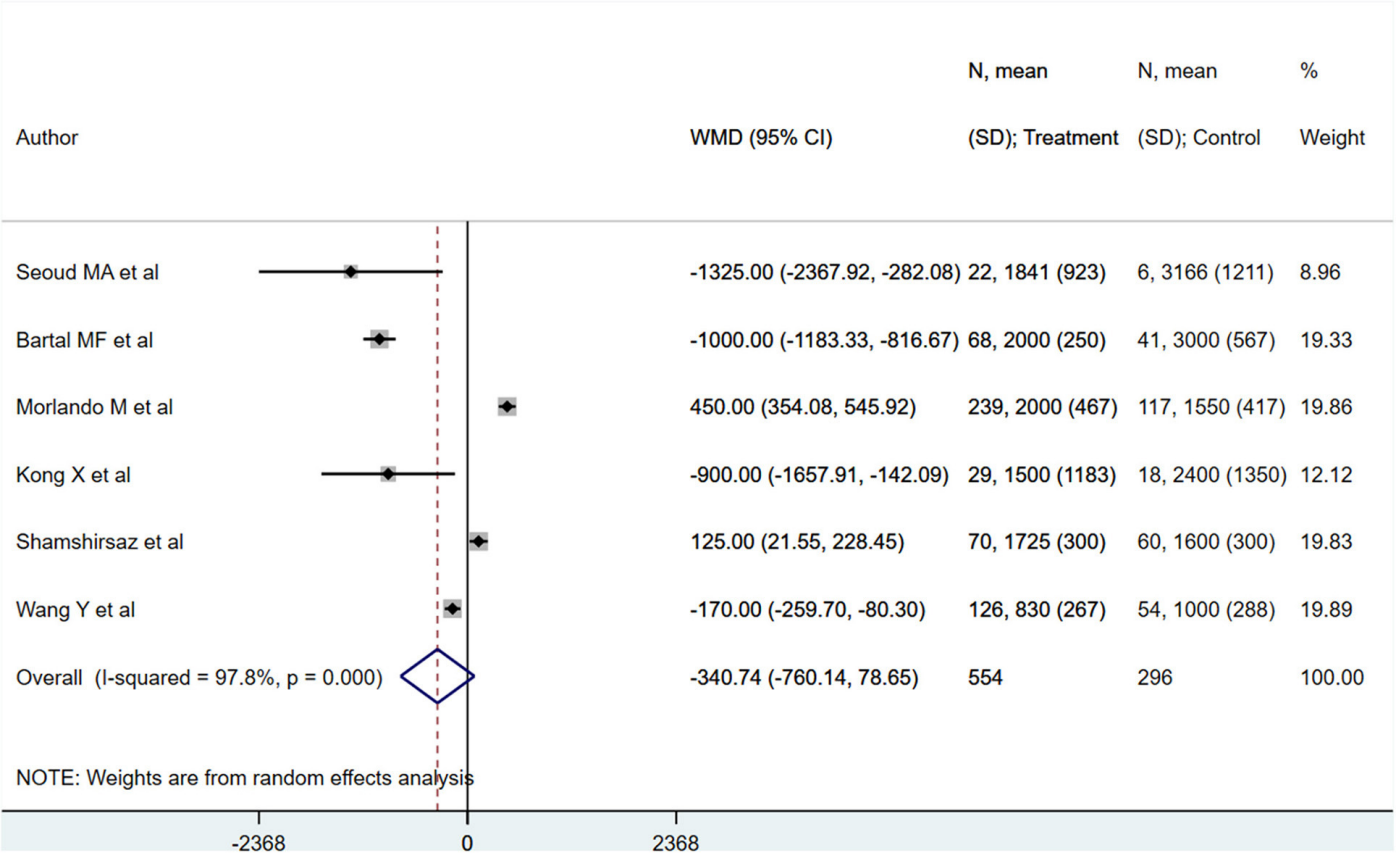
Maternal blood loss for PAS patients undergoing planned or emergency delivery.

PAS patients with planned deliveries had increased gestational ages (in weeks) [WMD 2.32; 95% CI: 1.60, 3.04] (Figure 2) compared to PAS patients who had emergent delivery. Egger's test did not indicate publication bias (*P* = 0.68). Those with planned deliveries also showed comparatively lower blood loss (in ml), although the difference was not statistically significant [WMD −340.7; 95% CI: −760.1, 78.6] (Figure 3). Egger's test did not indicate publication bias (*P* = 0.26).

**Figure 4:**
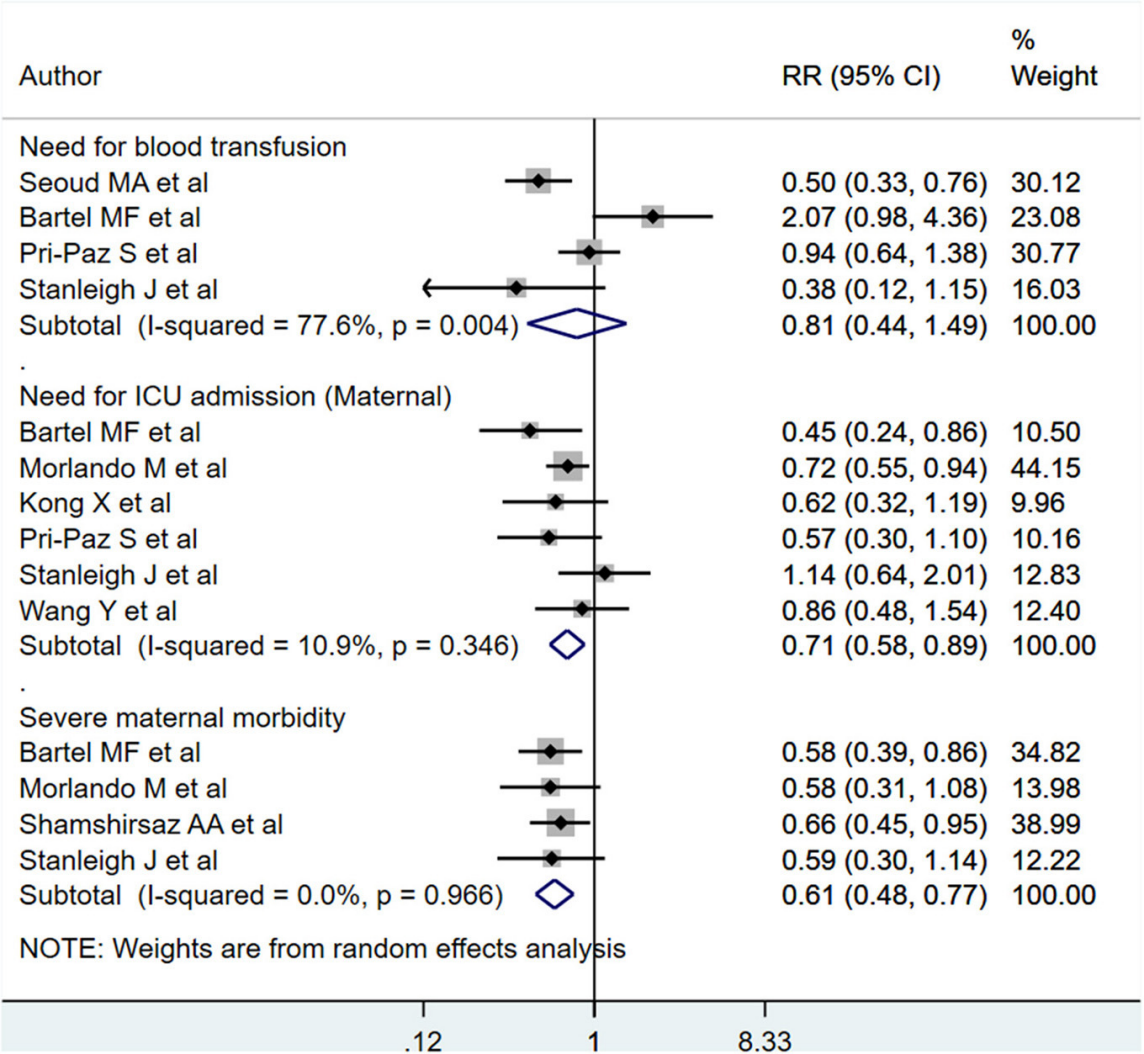
Maternal blood transfusion incidence, ICU admission, and severe morbidity for PAS patients undergoing planned or emergency delivery.

**Figure 5:**
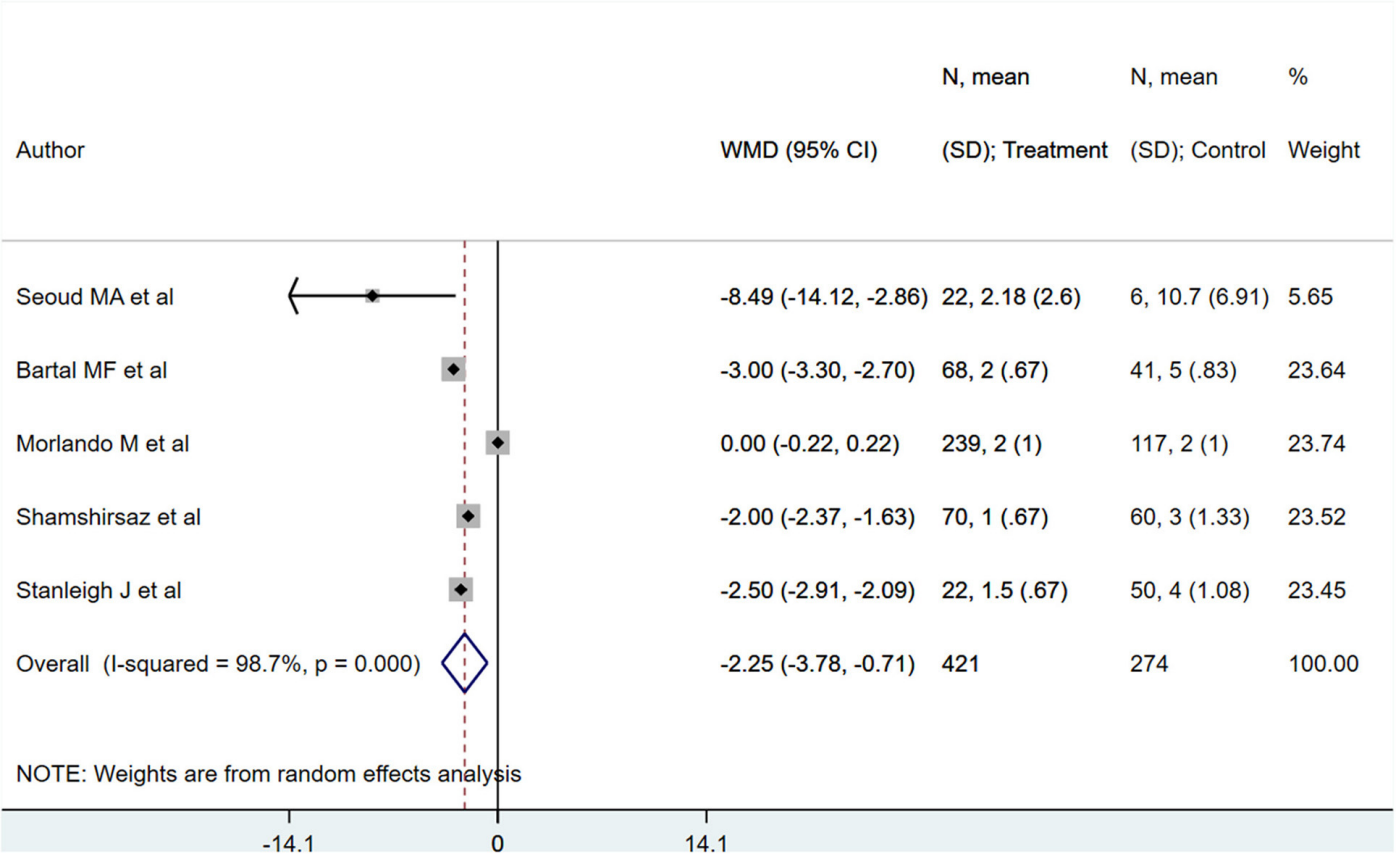
Units of blood transfused for PAS patients undergoing planned or emergency delivery.

**Figure 6:**
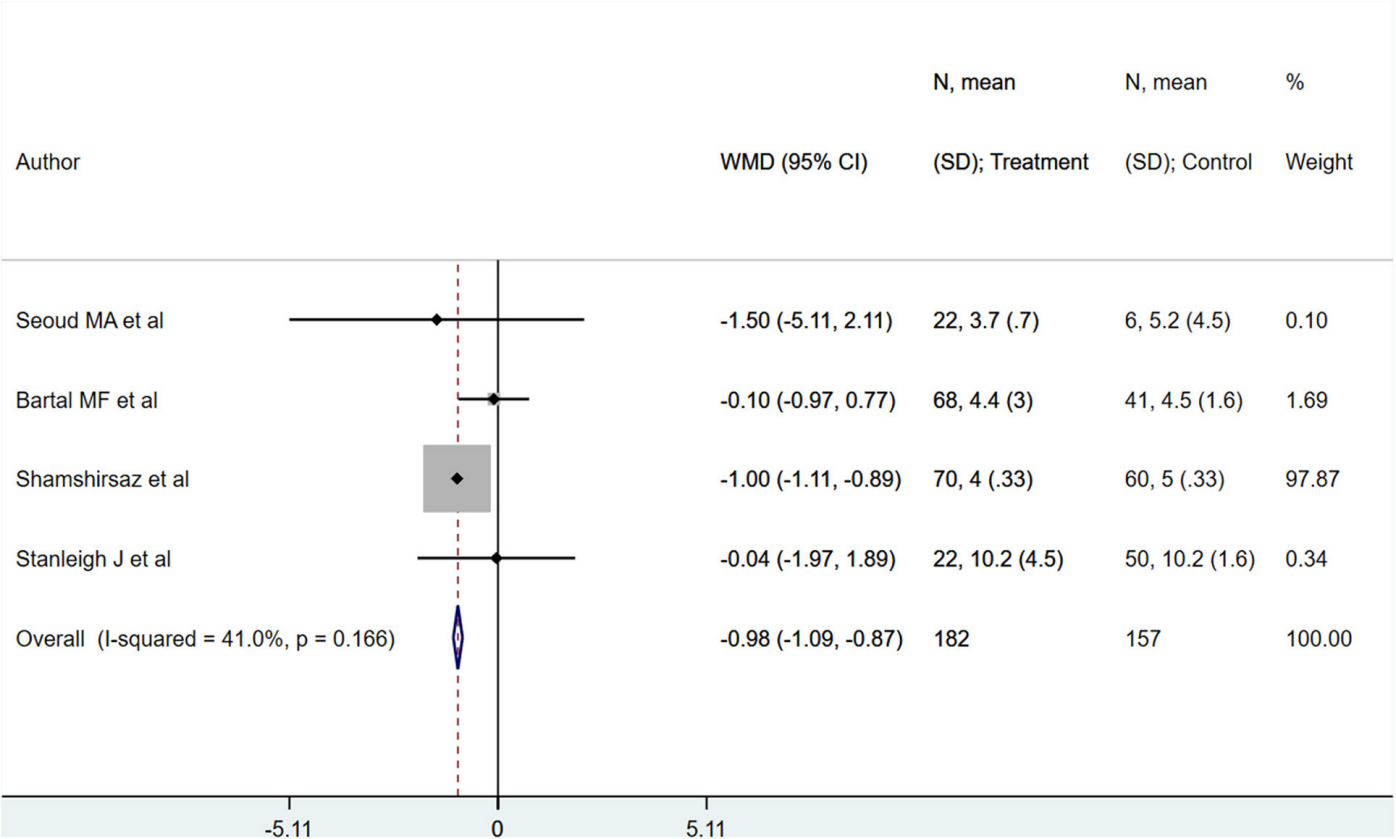
Duration of hospital stay for PAS patients undergoing planned or emergency delivery.

PAS patients with planned deliveries, relative to PAS patients with emergent deliveries, had decreased risks for maternal intensive care unit admission [RR 0.71; 95% CI: 0.58, 0.89] and severe maternal morbidity [RR 0.61; 95% CI: 0.48, 0.77] (Figure 4). There was no difference in blood transfusion incidence [RR 0.81; 95% CI: 0.44, 1.49] between the two groups. Egger's test did not indicate publication bias (*P* = 0.18 for ICU admission risk, *P* = 0.45 for severe maternal morbidity, and *P* = 0.38 for need for blood transfusion incidence). However, PAS patients with planned deliveries required fewer units of blood during transfusion transfused [WMD −2.25; 95% CI: −3.78, −0.71] and had shorter hospital stay durations (in days) [WMD −0.98; 95% CI: −1.09, −0.87] (Figures 5, 6). Egger's test did not indicate publication bias (*P* = 0.66 for units of blood transfused, *P* = 0.21 for duration of hospital stay).

### Neonatal Outcomes

**Figure 7:**
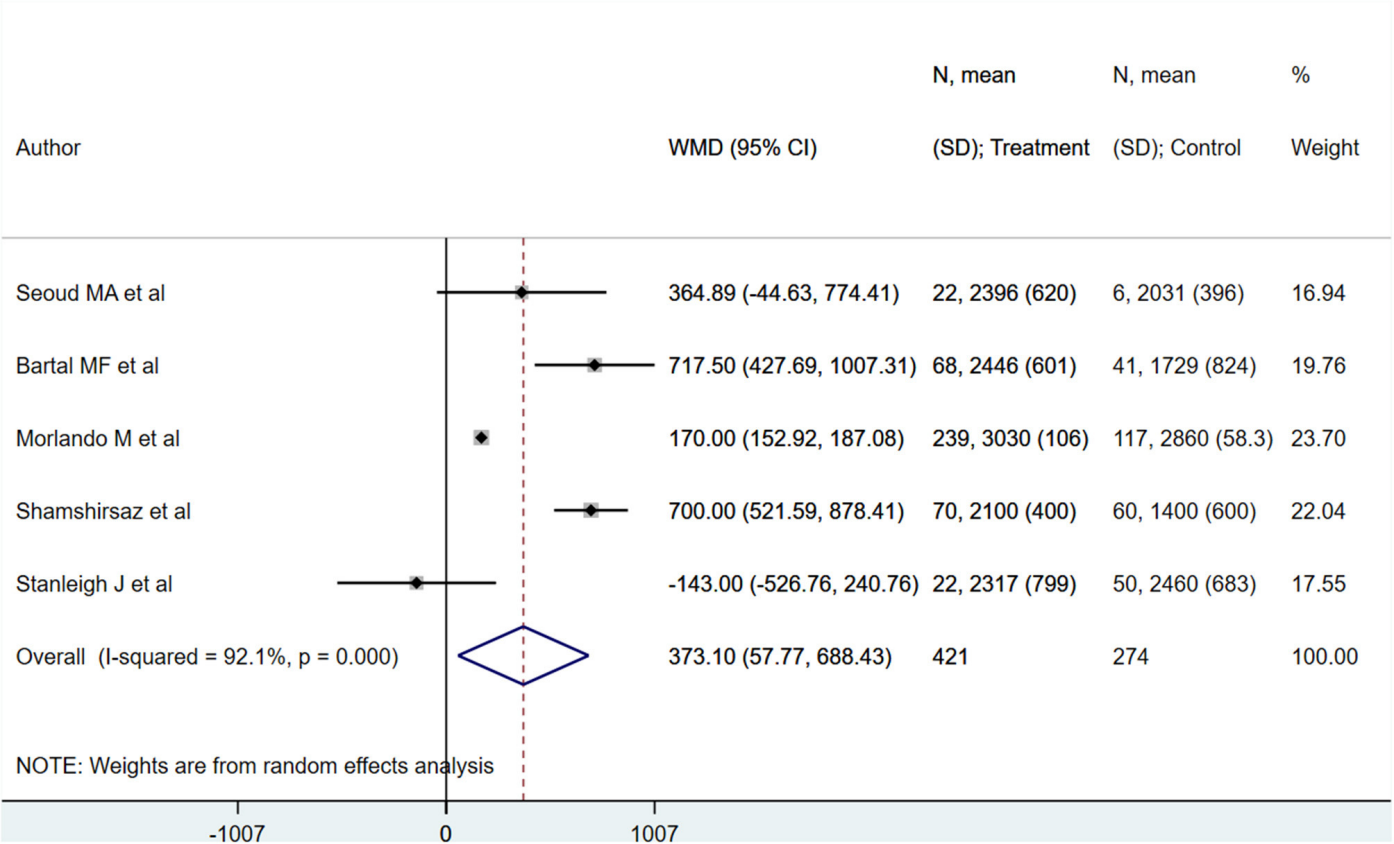
Neonatal weight for babies born to PAS patients *via* planned or emergency delivery.

**Figure 8:**
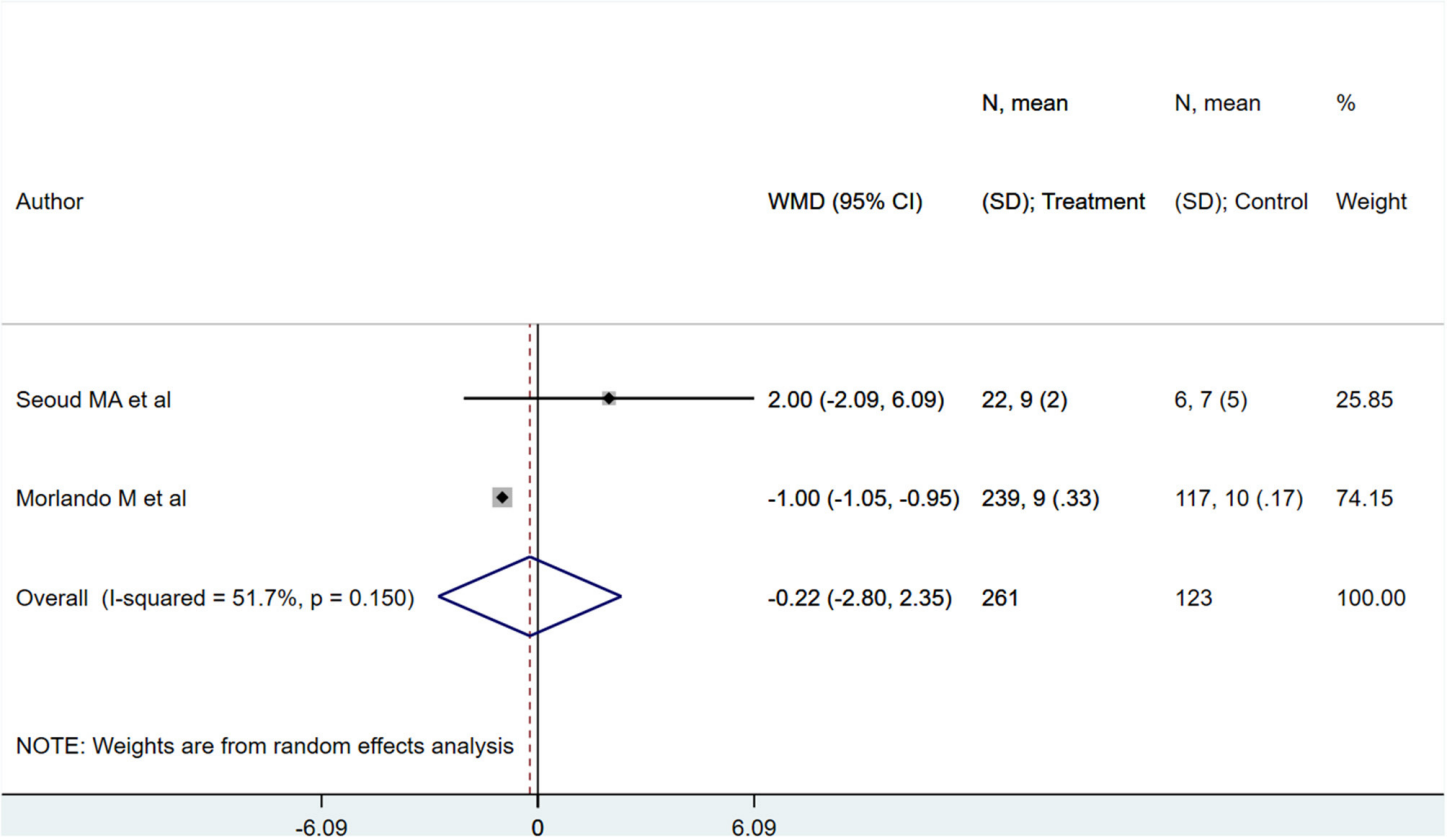
APGAR score at 5 min for babies born to PAS patients *via* planned or emergency delivery.

Neonates born to PAS patients *via* planned delivery weighed more than neonates born *via* emergent delivery (in grams) [WMD 373.10; 95% CI: 57.77, 688.43] (Figure 7). However, 5-min APGAR scores were not different between groups [WMD −0.22; 95% CI: −2.80, 2.35] (Figure 8). Egger's test did not indicate publication bias (*P* = 0.22 for neonatal weight, *P* = 0.49 for APGAR score).

**Figure 9:**
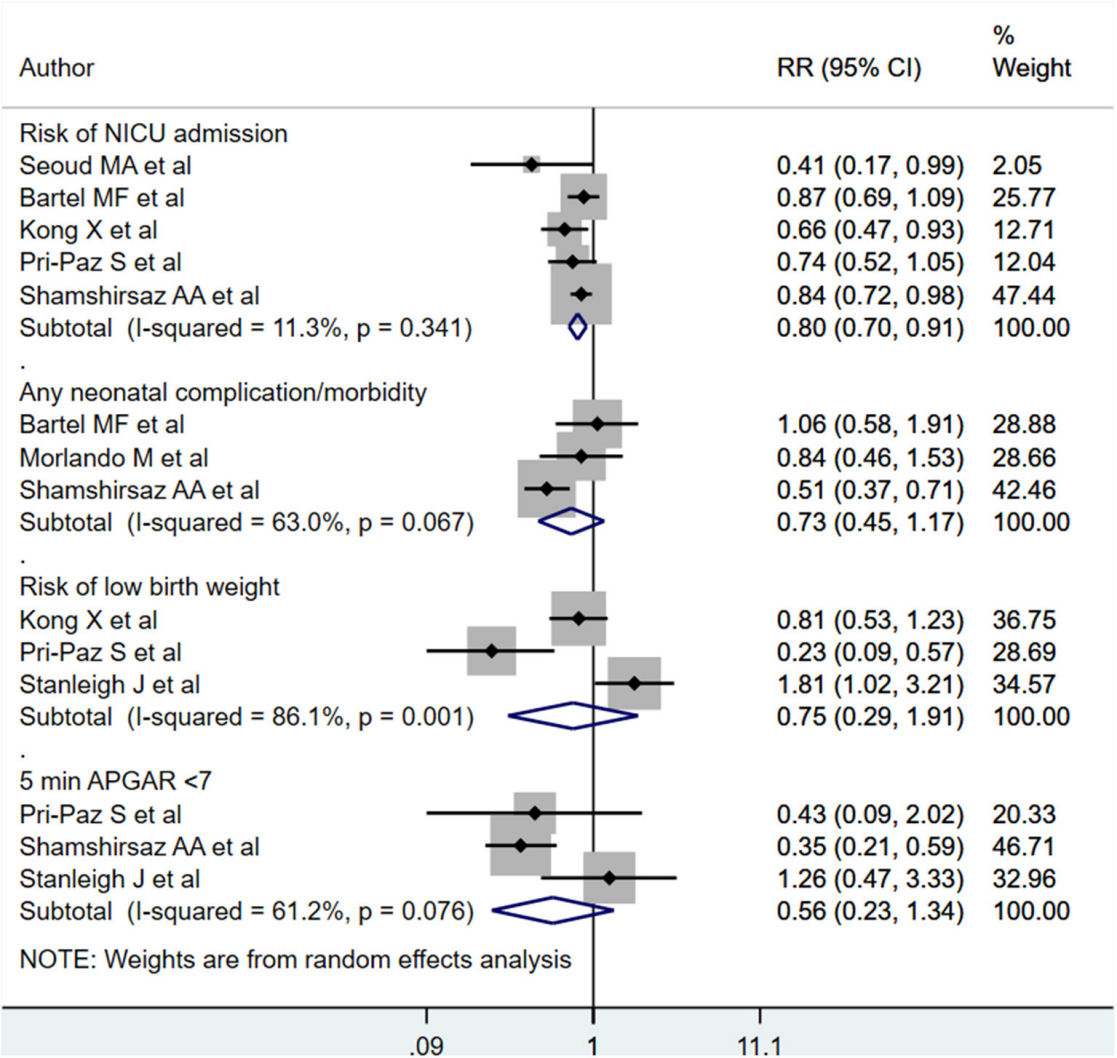
Risk of NICU admission, neonatal complications, low birth weight, and low APGAR score for babies born to PAS patients *via* planned or emergency delivery.

Neonates born to PAS patients *via* planned delivery had lower risk for NICU admission compared to neonates born *via* emergent delivery [RR 0.80; 95% CI: 0.70, 0.91] (Figure 9). No differences were noted for general complication/morbidity risk [RR 0.73; 95% CI: 0.45, 1.17], low birth weight [RR 0.75; 95% CI: 0.29, 1.91], or 5-min APGAR scores of under 7 [RR 0.56; 95% CI: 0.23, 1.32] (Figure 9). Egger's test did not indicate publication bias (*P* = 0.12 for admission to NICU, *P* = 0.31 for neonatal complications, P=0.71 for low birth weight, *P* = 0.23 for low APGAR score).

## Discussion

The current meta-analysis was conducted to compare the maternal and neonatal outcomes among women with placenta accrete spectrum (PAS), delivered through a planned approach and those with emergency delivery. The findings suggest that compared to mothers who had emergency delivery, those with planned delivery had increased gestational age, lesser units of blood transfused, lesser duration of hospital stay (in days), reduced risk for maternal admission to intensive care unit (ICU) and reduced risk of severe maternal morbidity. Although it did not reach statistical significance, the amount of blood loss was also seemingly higher in those with urgent/emergency delivery. Neonatal outcomes were also favorable in those with planned delivery as neonates born to such mothers had increased weight and lower risk for admission to NICU.

It is possible that the effect on neonatal weight is mediated through increase in gestational age. Further, it is well-documented that neonates born preterm have increased risk of being admitted to NICU and therefore, the reduced risk of admission to NICU in neonates born through planned delivery could be due to improved gestational age at delivery (26, 27). Increased blood loss in PAS has been previously documented and it due to invasion of placental trophoblast into the uterine myometrium or adjacent pelvic organs. Usually after delivery, placenta detaches from the uterus but in PAS, whole or part of placenta remains attached and any manipulation leads to severe bleeding and maternal morbidity. Findings of previous studies have substantiated this and documented an increased blood loss and units of blood transfused when placental removal was attempted, compared to cesarean hysterectomy in women with placenta accreta (28, 29).

While PAS is globally recognized as a high-risk obstetric condition, yet there is no universal consensus on managing a pregnant woman with this condition. Due to of a lack of randomized clinical trials, the optimal management of PAS disorders remains undefined and is determined by the capacity to diagnose invasive placentation preoperatively, depth of villous invasion, presenting symptoms and availability of local expertise. It is also important that the clinical diagnosis of PAS is made at the earliest or the patient is thoroughly investigated even with slightest suspicion. A recent study from Italy showed that antenatal suspicion of PAS was associated with improved maternal outcomes likely because of their referral to specialized centers for management (30). The findings of the meta-analysis reiterate that because of the risks associated with PAS, a planned delivery consisting of optimal diagnostic evaluation and involving a multidisciplinary team at a tertiary care or level 3/4 health care center is essential. Such a multidisciplinary team should essentially have an experienced obstetrician, neonatologist and critical care team of anesthetists, transfusion experts, and a radiologist trained in intervention radiology. A recent study by Sun et al. (31) offers an interesting and novel method to follow up and manage patients with PAS. The authors published a study wherein they compared the maternal and neonatal outcomes for patients with PAS between online-to-offline management model with standard care model. The authors used a social media platform where patients diagnosed with PAS could connect with the treating doctor and consult the doctor if adverse symptoms occurred. In the standard care model, once the diagnosis of PAS was made, the patients were instructed to go home to monitor by themselves and in case of hemorrhage or adverse symptom, the patients were instructed to go to hospital for emergent medical care. The authors noted that pregnant women managed by online-to-offline care model had reduced risk of hysterectomy, shorter hospital stay, and shorter response time from admission to emergency cesarean section. There is a need for more such novel methodologies to identify early signs of complications in patients with PAS.

This is probably the first attempt to pool the findings of the available studies comparing maternal and neonatal outcomes in women with PAS by planned and emergent delivery. There are some limitations of this meta-analysis. Majority of the studies included in the review were retrospective cohort based and therefore, the possibility of bias due to lack of adjustment for confounders cannot be ruled out. Further, there was a high degree of heterogeneity noted for some of the outcomes. We had adopted the random effects model to address the issue of heterogeneity. The reason for high heterogeneity could be the differences in the operational definitions used, methods to ascertain the outcomes such as total blood loss and the reliability of the diagnosis of PAS, i.e., the possibility of a false positive diagnosis for PAS on ultrasonography cannot be ruled out.

## Conclusion

PAS patients undergoing planned deliveries presented better maternal and neonatal outcomes compared to patients undergoing emergency delivery. This meta-analysis indicates that PAS management should be pre-planned with a team of experts consisting of obstetricians, pediatricians, intensive care specialists, transfusion specialists, and radiologists. To enable this, early diagnosis of PAS through ultrasonography is important.

## Data Availability Statement

The raw data supporting the conclusions of this article will be made available by the authors, without undue reservation.

## Author Contributions

WZ and FZ: conceived and designed the study and wrote the paper. SL, JC, FH, JX, and MY: literature search and data collection. JX and MY: analyzed the data. SL, JC, and FH: reviewed and edited the manuscript. All authors read and approved the final manuscript.

## Conflict of Interest

The authors declare that the research was conducted in the absence of any commercial or financial relationships that could be construed as a potential conflict of interest.

## Publisher's Note

All claims expressed in this article are solely those of the authors and do not necessarily represent those of their affiliated organizations, or those of the publisher, the editors and the reviewers. Any product that may be evaluated in this article, or claim that may be made by its manufacturer, is not guaranteed or endorsed by the publisher.
