# Supplementary material for: Maternal and Neonatal Outcomes After Planned or Emergency Delivery for Placenta Accreta Spectrum: A Systematic Review and Meta-Analysis
Source: Front Med (Lausanne). 2021 Sep 28;8:731412. doi: 10.3389/fmed.2021.731412 (PMC8505704; doi:10.3389/fmed.2021.731412)
Supplement: Supplementary file 1 [file Table_1.DOC]

**Supplemental table 1. Search strategy for identifying eligible studies for inclusion**

| **Search strategy**  #1 (placenta accreta OR placenta increta OR placenta percreta OR placental anomalies)  #2 (maternal outcomes OR neonatal outcomes OR perinatal outcomes OR intrapartum outcomes OR pregnancy outcomes OR infant outcomes)  #3 (planned delivery OR emergency delivery OR emergent delivery OR elective delivery)  #4 (#1 AND #2 AND #3)  #5 (Addresses[ptyp] OR Autobiography[ptyp] OR Bibliography[ptyp] OR Biography[ptyp] OR pubmed books[filter] OR Case Reports[ptyp] OR Congresses[ptyp] OR Consensus Development Conference[ptyp] OR Directory[ptyp] OR Duplicate Publication[ptyp] OR Editorial[ptyp] OR Systematic reviews OR Meta analysis OR Festschrift[ptyp] OR Guideline[ptyp] OR In Vitro[ptyp] OR Interview[ptyp] OR Lectures [ptyp] OR Legal Cases[ptyp] OR News[ptyp] OR Newspaper Article[ptyp] OR Personal Narratives [ptyp] OR Portraits[ptyp] OR Retracted Publication[ ptyp] OR Twin Study[ptyp] OR Video-Audio Media[ptyp])  #6 (#4 NOT #5) |
| --- |

**Supplementary table 2. Study quality assessments using the adapted Ottawa-Newcastle Risk of Bias Assessment tool**

|  | Seoud MA et al | Bartal MF et al | Kong X et al | Morlando M et al | Pri-Paz S et al | Shamshirsaz AA et al | Stanleigh J et al | Wang Y et al | Meller CH et al |
| --- | --- | --- | --- | --- | --- | --- | --- | --- | --- |
| Representativeness/appropriateness of participant selection  Random or consecutive recruitment=Y  Convenience sample=N  Not reported or unclear | Y | Y | Y | Y | Y | Y | Y | Y | Y |
| Control for baseline differences in cohorts  Similarity of groups at baseline or adjustment in analyses=Y  No attempt to control or adjust=N  Not reported=NR | Y | Y | Y | Y | Y | Y | Y | Y | Y |
| Loss to follow-up  Explanation provided for loss of participants and/or intention to treat=Y  No explanation =N | Y | N | Y | Y | Y | N | Y | Y | N |
| Masking of exposure to outcomes assessor  Description of masking=Y  No masking or no description =N | Y | Y | Y | Y | Y | Y | Y | Y | N |
| Ascertainment of condition  Description of ascertainment/diagnostic criteria=Y  No description or patient self-report=N | Y | Y | Y | Y | Y | Y | Y | Y | Y |
| Documentation of other treatment modalities  Documentation=Y  No documentation=N | N | Y | Y | Y | Y | Y | N | N | Y |
| Extent to which valid outcomes are described  Adequate description of outcome=Y  Insufficient detail regarding outcome or follow-up time=N | Y | Y | Y | Y | Y | N | Y | Y | Y |
| Prespecification of harms, mode of harms collection  Description of a list of harms assessed or monitoring=Y  No such description or passive harms collection=N  No adverse events reported=NA | Y | N | Y | Y | Y | N | Y | Y | N |
| Financial Conflict of interest (COI)  Funding source reported=Y  Funding source not reported=N | Y | Y | Y | N | Y | N | Y | Y | Y |
